# Scrub Typhus Combined With Septic Shock Disseminated Intravascular Coagulation and Significant Hyperfibrinolysis: A Case Report and Review of the Literature

**DOI:** 10.1155/crdi/1931423

**Published:** 2025-03-04

**Authors:** Dewen Ma, Xiaohong Wan, Haihui Yang, Liying Yang, Ankang Peng, Quping Yuan, You Li, Shunhang Xu

**Affiliations:** ^1^Intensive Care Unit, People's Hospital, Pu'er City, Yunnan, China; ^2^Intensive Care Unit, The Second Affiliated Hospital of Kunming Medical University, Kunming, Yunnan, China; ^3^Department of Cardiology, People's Hospital, Pu'er City, Yunnan, China; ^4^Department of Pediatrics, People's Hospital, Pu'er City, Yunnan, China

## Abstract

**Introduction:** Scrub typhus is an acute infectious disease caused by *Orientia tsutsugamushi*, whose pathophysiology is characterized by systemic small-vessel vasculitis. Its high misdiagnosis rate stems from its nonspecific clinical features. If not diagnosed and treated in time, patients may rapidly progress to multiorgan dysfunction syndrome (MODS) or even disseminated intravascular coagulation (DIC), posing a severe threat to life.

**Case Presentation:** The patient was a 68-year-old male with “recurrent fever and dry cough for six days.” He was admitted to the hospital with a diagnosis of scrub typhus. After admission, he developed severe acute respiratory distress syndrome (ARDS), MODS, septic shock, DIC with thrombocytopenia, hypofibrinogenemia, significant hyperfibrinolysis, and myocardial depression. The patient improved following treatment with doxycycline, moxifloxacin, renal replacement therapy, blood transfusion, antifibrinolysis, invasive mechanical ventilation, and other supportive therapies. The patient's coagulation profile in DIC caused by scrub typhus demonstrated significant hyperfibrinolysis, differing from that of garden-variety sepsis, and no similar cases were identified in a search of medical literature/databases.

**Conclusion:** The fibrinolytic system in DIC caused by scrub typhus is excessively active, and antifibrinolytic therapy may benefit such patients. Further research on the distinct coagulation abnormalities in scrub typhus–associated DIC would be highly valuable compared to sepsis-associated DIC.

## 1. Introduction

Scrub typhus is a rapidly spreading infectious disease caused by *Orientia tsutsugamushi*. It is a naturally occurring epidemic disease, with rodents serving as the primary source of infection and chigger larvae acting as the carrier [1]. The condition is characterized by systemic small-vessel vasculitis, which can lead to multiorgan damage. In addition, the release of toxins from the pathogen upon its death may result in clinical indications of toxemia. Scrub typhus poses a significant public health challenge in the Asia-Pacific region, threatening the lives and well-being of over a billion people globally each year [2].

The fatality rate of scrub typhus remains concerning. The median incidence of mortality is approximately 6% for untreated patients and 1.4% for treated patients; however, without timely and appropriate treatment, the fatality rate can reach as high as 70% [3, 4].

Several studies report that misdiagnosis rates for scrub typhus can be as high as 75%, often due to its nonspecific clinical presentation. Delayed or inappropriate therapy frequently results in serious complications or sequelae [5]. For example, Emily and her colleagues conducted a retrospective analysis of 11,535 patients with scrub typhus infections. They found the following complications: hepatitis (40.5% of cases), thrombocytopenia (28.4%), acute respiratory distress syndrome (ARDS) (20.5%), acute kidney injury (19.2%), meningitis (16.4%), shock (16.2%), and myocarditis (15.5%). Of these, multiorgan dysfunction syndrome (MODS) accounted for 17.4% [6]. Kamath, Kumari, and Sunder reported that scrub typhus complicated with disseminated intravascular coagulation (DIC) occurs in approximately 2.4% of cases [7].

Though coagulopathy has been described in scrub typhus, the specific mechanisms underlying the abnormalities in the coagulation and fibrinolysis pathways remain poorly elucidated. Hence, we decided to report a case of hyperfibrinolysis in scrub typhus–associated DIC.

## 2. Case Report

**Figure 1:**
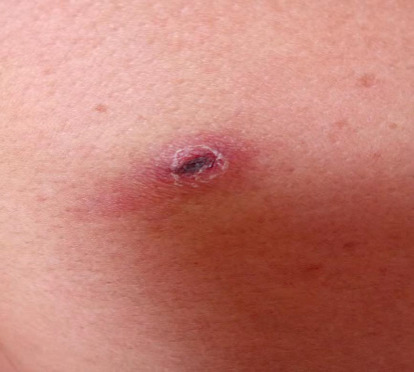
A 0.5 ∗ 0.5 cm crust is visible on the patient's left inner thigh.

A 68-year-old male was admitted to the intensive care unit (ICU) with “recurrent fever and dry cough for six days.” Upon examination, a 0.5 × 0.5 cm crusted lesion was observed on the left inner thigh (Figure 1). The patient's initial vital signs were as follows: temperature: 39.3°C, heart rate: 110 beats/min, respiratory rate: 25 breaths/min, blood pressure: 80/48 mmHg, and SpO_2_: 89%.

### 2.1. Laboratory Findings

Arterial blood gas analysis revealed a pH of 7.3, PCO_2_ of 29 mmHg, PO_2_ of 76 mmHg, FiO_2_ of 100%, lactate (Lac) of 2.9 mmol/L, and HCO_3_- of 14.1 mmol/L. Laboratory findings upon admission are summarized in Table 1.

The Weil–Felix test was positive, with a titer of 1:171, supporting the diagnosis of scrub typhus.

### 2.2. Imaging Studies

Abdominal ultrasound revealed a thickened gallbladder wall, a mildly enlarged spleen, and slightly enhanced echogenicity of both renal parenchymas, with urinary salt crystallization in the right kidney and bilateral pleural effusion.

**Figure 2:**
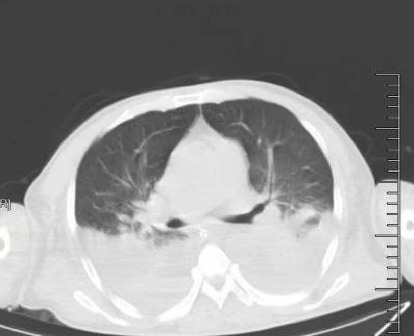
Chest CT: bilateral pleural cavities with a moderate amount of effusion and incomplete expansion of adjacent lung tissue.

Chest CT showed bilateral pleural effusions with incomplete expansion of adjacent lung tissue (Figure 2).

### 2.3. Diagnosis

The patient was diagnosed with scrub typhus, MODS, DIC, septic shock, pulmonary infection, severe ARDS, respiratory failure, acute renal insufficiency, hepatic injury, and hypofibrinogenemia based on clinical symptoms, laboratory results, imaging findings, and the ISTH diagnostic criteria for DIC (Table 2). He was further confirmed to have an *Orientia tsutsugamushi* infection accompanied by pneumonia and septic shock.

### 2.4. Treatment Plan and Progress

**Figure 3:**
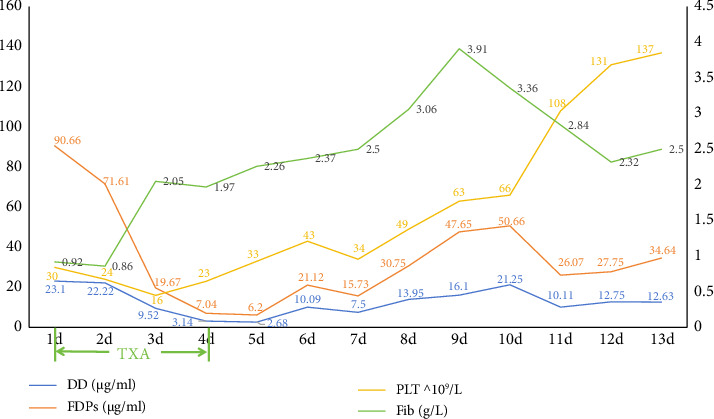
After antifibrinolytic therapy, the patient's plasma fibrin (pro) degradation products and D-dimer levels gradually decreased, while fibrinogen levels returned to normal on the third day. Under comprehensive treatment, including anti-infective therapy, antifibrinolytic therapy, and supportive measures, platelet counts progressively increased and reached normal levels by the fourth day.

**Figure 4:**
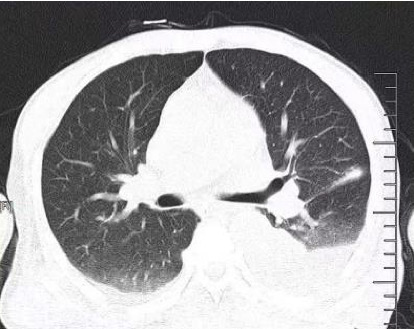
After anti-infective treatment and fiberoptic bronchoscopic alveolar lavage, the patient's lung-infected lesions were significantly resorbed compared to before.

**Figure 5:**
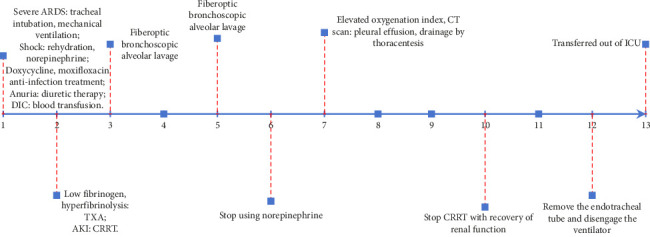
This timeline illustrates the patient's clinical course after ICU admission. On Day 6, norepinephrine was discontinued as the patient's shock resolved. By Day 10, renal function improved significantly, leading to the termination of CRRT. On Day 12, the tracheal tube was extubated following recovery of pulmonary function, and the patient was transferred out of the ICU on Day 13. The patient was discharged from the hospital on Day 31 after full recovery, and no residual symptoms were observed during the follow-up.

The treatment plan included a combination of doxycycline and moxifloxacin for anti-infective therapy, active rehydration, and norepinephrine to address the shock. The patient exhibited Type 1 respiratory failure and metabolic acidosis and was administered invasive mechanical ventilation. The patient had anuria, and despite receiving vigorous diuretic treatment, the condition persisted. Consequently, the patient was administered continuous renal replacement therapy (CRRT). The patient was diagnosed with DIC with hypothrombinemia and coagulopathy. The treatment involved administering an active blood transfusion to replenish the deficient coagulation components. The patient exhibited a marked hyperfibrinolytic state, with plasma fibrin degradation products measuring 90.66 *μ*g/mL and D-dimer levels at 23.1 *μ*g/mL. In addition, the patient experienced severe hypofibrinogenemia, with a fibrinogen level of 0.92 g/L. Although there is limited evidence suggesting that high doses or prolonged use of steroids may contribute to hypofibrinogenemia through the suppression of acute-phase reactants, the low fibrinogen level, in this case, was primarily attributed to the hyperfibrinolytic state associated with scrub typhus–related DIC. To address this condition, the patient received a monocyclic acid antifibrinolytic medication. After administering antifibrinolytic medication, the patient's plasma fibrin degradation products and D-dimer levels steadily declined, while fiber levels returned to normal by the third day. The patient was diagnosed with DIC. Anticoagulation was not administered, and platelet levels progressively rose until reaching normal levels on the fourth day of antifibrinolytic medication (Figure 3). Following intensive treatment, the patient's shock was successfully resolved, and norepinephrine was stopped on the sixth day after admission. The patient's renal function steadily improved, and CRRT was terminated on the tenth day. The patient's lung function gradually improved after aggressive anti-infective therapy and periodic fiberoptic bronchoscopic alveolar lavage (Figure 4). The tracheal tube was removed on the 12^th^ day. The patient was discharged from the ICU on the 13^th^ day (Figure 5). The patient was released from the hospital on the 31^st^ day. During our 6-month follow-up appointment, the patient was healthy without any lingering effects.

## 3. Discussion

Scrub typhus, caused by the obligate intracellular bacterium *Orientia tsutsugamushi*, is distinct from other rickettsial diseases due to its unique genetic makeup and cell wall structure. *Orientia tsutsugamushi* is a Gram-negative bacterium exclusively residing within host cells [8]. Unlike other Gram-negative bacteria, it lacks lipopolysaccharide and exhibits minimal nonclassical peptidoglycan expression [9]. Endothelial cells serve as primary targets for *Orientia tsutsugamushi* during systemic infection, with the bacteria localizing within the endothelial cells of various organs, including the heart, lungs, brain, liver, kidneys, pancreas, and skin. The infection induces endothelial cell activation and apoptosis, facilitating leukocyte adhesion, transportation, antigen presentation, and cytokine production [10]. Studies have shown elevated levels of TNF-α in patients with scrub typhus, correlating with disease severity and mortality risk [11]. TNF-α contributes to endothelial dysfunction by damaging the polysaccharide envelope, increasing the expression of adhesion molecules, and promoting apoptosis [12, 13], ultimately leading to DIC. In a study by Kamath, Kumari, and Sunder, the mortality rate of scrub typhus was reported to be 6.3% [7]. This rate underscores the potential severity of the infection and the importance of timely and effective treatment.

In sepsis, inflammatory mediators such as TNF-α and IL-6 enhance TF expression on endothelial cells, promoting thrombosis. However, the fibrinolytic system in sepsis is often suppressed due to elevated levels of plasminogen activator inhibitor-1 (PAI-1), which inhibits fibrinolysis and promotes microthrombus persistence [14, 15]. In contrast, in this case, the patient with scrub typhus exhibited a hyperactive fibrinolytic system, which facilitated clot breakdown and restored microvascular patency. This difference may partly explain why scrub typhus has a lower mortality rate (6.3%) compared to severe sepsis despite both conditions involving endothelial damage and systemic inflammation [16].

Antifibrinolytic therapy with tranexamic acid was administered to address the hyperfibrinolytic state. Although D-dimer and FDP levels remained elevated, the patient's fibrinogen levels progressively normalized, and platelet counts improved after treatment (Figure 3). This suggests that antifibrinolytic therapy may reduce fibrinogen and platelet consumption by slowing excessive clot breakdown. However, further studies are needed to evaluate the safety and efficacy of antifibrinolytic agents in scrub typhus–associated DIC, as their use may theoretically increase the risk of thrombotic complications [17].

We searched PubMed and Google Scholar to identify studies on DIC and coagulation abnormalities related to scrub typhus. Our search yielded 19 case reports on scrub typhus–associated DIC and two broader studies analyzing coagulation profiles in scrub typhus patients. However, none of these studies specifically addressed hyperfibrinolysis. For instance, Singla et al. observed elevated D-dimer levels (> 4 μg/mL) in three out of 71 scrub typhus patients but did not include detailed fibrinogen analysis [16]. Similarly, Lee et al. demonstrated higher D-dimer and FDP levels in scrub typhus patients than in healthy controls but did not investigate cases with DIC or hyperfibrinolysis [18]. The lack of detailed studies on scrub typhus–associated DIC, particularly involving hyperfibrinolysis, underscores the need for further investigation. This case may represent one of the first reports of significant hyperfibrinolysis in scrub typhus–associated DIC, highlighting a distinct coagulation profile that warrants more comprehensive studies.

## 4. Conclusion

This case report highlights a unique presentation of scrub typhus–associated DIC, characterized by significant hyperfibrinolysis. The use of antifibrinolytic therapy, in this case, provides new insights into managing scrub typhus–associated coagulation disorders. However, current literature lacks detailed descriptions of the distinct coagulation patterns observed in scrub typhus–associated DIC, particularly hyperfibrinolysis. While scrub typhus and sepsis involve endothelial dysfunction and systemic inflammation, their differing impacts on coagulation and fibrinolysis warrant further investigation. Future studies should aim to elucidate the underlying mechanisms of hyperfibrinolysis in scrub typhus and assess the role of antifibrinolytic agents in its treatment.

## Figures and Tables

**Table 1:** Laboratory findings upon admission.

Parameter	Patient value	Normal range
Platelet count (10^9^/L)	30	123–350
Leukocyte count (10^9^/L)	6.5	4–10
Neutrophil percentage (%)	83.1	50–70
Fibrinogen (g/L)	0.92	2–4
D-dimer (μg/mL)	23.1	< 0.55
Plasma FDP (μg/mL)	90.66	< 5
Total bilirubin (μmol/L)	23.9	< 21
Creatinine (μmol/L)	479	53–115
Urea nitrogen (mmol/L)	24.25	2.9–8.2
Aspartate aminotransferase (AST, IU/L)	230	< 40
Alanine aminotransferase (ALT, IU/L)	75	< 40
Albumin (g/L)	21.4	35–50

**Table 2:** ISTH overt DIC.

Item	Score	ISTH overt DIC range
Platelet count (× 109/L)	2	< 50
	1	≥ 50, < 100

FDP (D-dimer)	3	Strong increase
	2	Moderate increase

Prothrombin time	2	≥ 6 s
	1	≥ 3 s, < 6 s

Fibrinogen (g/L)	1	< 1
Total score for overt DIC	≥ 5	

*Note:* Diagnosis of overt DIC with a total score of ≥ 5.

Abbreviations: DIC, disseminated intravascular coagulation; ISTH, International Society on Thrombosis and Hemostasis.

## Data Availability

All data generated or analyzed during this study are included in the published article. If additional data are required, they are available from the corresponding author upon reasonable request.
